# Asthma Phenotypes in the Era of Personalized Medicine

**DOI:** 10.3390/jcm12196207

**Published:** 2023-09-26

**Authors:** Victor Gonzalez-Uribe, Sergio J. Romero-Tapia, Jose A. Castro-Rodriguez

**Affiliations:** 1 Alergia e Inmunología Clínica, Hospital Infantil de México Federico Gómez, Ciudad de Mexico 06720, Mexico; dr.victorgonzalezu@gmail.com; 2 Facultad Mexicana de Medicina, Universidad La Salle México, Ciudad de Mexico 14000, Mexico; 3 Health Sciences Academic Division (DACS), Universidad Juárez Autónoma de Tabasco, Villahermosa 86040, Mexico; sjrtapia@hotmail.com; 4 Department of Pediatric Pulmonology, School of Medicine, Pontificia Universidad Católica de Chile, Santiago 7820436, Chile

**Keywords:** asthma, precision medicine, phenotypes, endotypes, pathogenetic mechanism

## Abstract

Asthma is a widespread disease affecting approximately 300-million people globally. This condition leads to significant morbidity, mortality, and economic strain worldwide. Recent clinical and laboratory research advancements have illuminated the immunological factors contributing to asthma. As of now, asthma is understood to be a heterogeneous disease. Personalized medicine involves categorizing asthma by its endotypes, linking observable characteristics to specific immunological mechanisms. Identifying these endotypic mechanisms is paramount in accurately profiling patients and tailoring therapeutic approaches using innovative biological agents targeting distinct immune pathways. This article presents a synopsis of the key immunological mechanisms implicated in the pathogenesis and manifestation of the disease’s phenotypic traits and individualized treatments for severe asthma subtypes.

## 1. Introduction

It is now understood that asthma is a complex condition influenced by the interplay between environmental exposures and epigenetic regulations. Asthma is widely acknowledged as a multifaceted syndrome characterized by airway hyperreactivity triggered by various factors, each with a distinct pathobiology [1]. Clinically, this presents as symptoms such as cough, wheezing, and shortness of breath. The most-prevalent and consequently well-explored prototype is asthma linked to allergic sensitization mediated by T-helper Type 2 (Th2) cells, now termed Type 2 (T2) asthma [2]. Progress in managing T2 asthma has pinpointed a subgroup labeled “difficult-to-control” or “severe” asthma, which exhibits poor responsiveness to current therapies primarily designed for T2 inflammation patterns [2,3,4,5]. As a result, the emergence of disease heterogeneity in the era of personalized medicine has reemphasized the necessity for a more-comprehensive asthma definition. In this review, we delve into recent breakthroughs in our comprehension of asthma’s pathobiology, its phenotypes and endotypes, and their implications for the clinical effectiveness of targeted therapies employed in treating severe and uncontrolled asthma.

Asthma arises from pathogenesis involving the innate and adaptive immune systems and epithelial cells. The primary clinical symptoms encompass mucus overproduction, airway remodeling, and the development of bronchial blockage and hyperreactivity [6]. The various manifestations of asthma in its different “phenotypes” can be attributed to the intricate interplay among diverse immune pathways [7]. Historically, asthma linked to eosinophilic-mediated inflammation has been associated with the overexpression and activation of Th-2 cells. Moreover, research has unveiled a subtype known as neutrophilic asthma involving T-helper Type 17 (Th-17) cells [8]. An intriguing discovery emerged when the capability of Type 2 innate lymphoid cells (ILC-2) and basophils to trigger eosinophilic inflammation in specific groups of asthmatic patients came to light [9]. This finding underscores the remarkable diversity inherent in asthma. This perspective gained support from cytokine-targeted therapy clinical studies showcasing symptom reduction in patients [10,11,12,13,14,15]. Consequently, our objective shifted towards achieving a more-comprehensive understanding of the numerous illness categories to offer a safer, more-precise, and more-potent approach to treatment.

## 2. Pathogenetic Mechanism

In the past, asthmatic airway inflammation was solely considered a facet of the immune system’s adaptive response. Nevertheless, the discovery of ILC-2 and its potential implication in atopic conditions [16] has provided a more lucid understanding of innate immunity’s vital role in shaping the inflammatory response that sets apart different asthma phenotypes [17].

Innate lymphoid cells (ILCs) are a group of lymphocytes devoid of lineage-specific markers or antigen receptors seen on myeloid, dendritic, B, or T cells. These ILCs comprise three distinct subsets known thus far: Group 1 ILCs (ILC-1), which generate interferon-gamma (IFN-γ), ILC-2, which produce cytokines traditionally associated with Th-2 cells (IL-4, IL-5, IL-13), and ILC-3, which yield IL-17 and/or IL-22 [5,18]. ILC-2 are vital in rapid inflammatory responses to helminthic and viral infections [19]. Lacking antigen-specific receptors, these cells are pivotal in coordinating airway inflammation in eosinophilic asthma phenotypes, irrespective of atopic status [16]. However, they respond to signals originating from epithelium, mediated by “alarmins” (such as thymic stromal lymphopoietin (TSLP), IL-25, and IL-33) secreted by epithelial cells [20]. TSLP, a cytokine of the IL-2 family, is produced by various cell types, including lung epithelial cells [21]. TSLP significantly contributes to the mediation of corticosteroid resistance associated with ILC-2-induced airway inflammation, and it triggers the upregulation of adaptive immune responses in asthma [22]. TSLP binds to a heterodimeric surface receptor expressed on a variety of cells, including T cells, B cells, natural killer (NK) cells, monocytes, basophils, eosinophils, ILC-2, dendritic cells, mast cells (MCs), and epithelial cells [23,24,25].

Interleukin 33 (IL-33), a member of the IL-1 family of pro-inflammatory cytokines, features an IL-1-like domain on one of its terminal chains [26]. This structural characteristic allows IL-33 to bind to the ST2 receptor, also known as the Interleukin 1-like receptor—a member of the Toll-like receptor family expressed on numerous immune cells, including Th-2 and ILC-2 [27].

**Figure 1:**
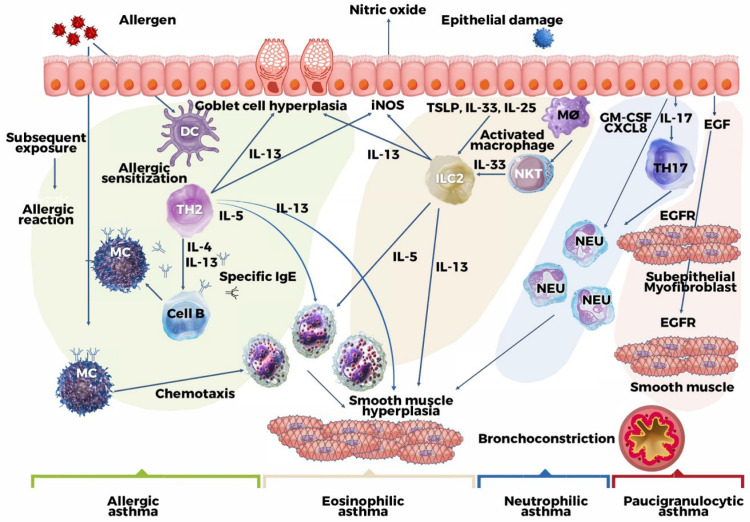
The distinct asthma endotypes. The green and yellow backgrounds of Type 2 (T2) inflammation endotypes correspond to allergic and non-allergic eosinophilic asthma, respectively. Non-T2 variants have blue and pink backgrounds and refer to neutrophilic asthma and asthma with minimal inflammation. For those phenotypes, the mechanism is associated with molecules that promote the proliferation and activation of myofibroblasts and smooth muscle cells. iNOS = inducible nitric oxide synthase, TSLP = stromal lymphopoietin thymic, GM-CSF = granulocyte-macrophage colony-stimulating factor, DC = dendritic cell, MC = mast cell, CXCL8 = C-X-C chemokine ligand 8 motif, EGFR = EGF receptor, EGF = epidermal growth factor, Eos = eosinophil, FeNO = fractional exhaled nitric oxide, IL = Interleukin, Neu = neutrophil, M∅ = macrophage, NKT = natural killer T cell. Adapted from Godar M. et al. [17].

The third element in the “alarmins” trio is Interleukin 25 (IL-25). This cytokine enhances the subsequent cascade of pro-inflammatory mediators: IL-25 secretion is governed by epithelial cell damage, which, in turn, is influenced by exposure to proteases from exogenous antigens [28]. Furthermore, IL-25 activates the NF-kB signaling pathway by activating the signaling cascade. This prompts the secretion of Th2 cytokines IL-4, IL-5, and IL-13, triggering the Type 2 inflammatory response [29] (Figure 1).

Historically, the immunopathology of asthma was depicted as a linear progression that differentiated between innate and adaptive immunity. Although this theory prompted researchers to deviate from the conventional cascade, it did enable the differentiation of the inflammatory response mediated by innate and adaptive immunity in asthma. Despite this distinction, the pathogenesis of asthma adheres to a shared cascade, with signaling molecules common to both immune pathways [5,30]. Regarding the roles of Th-2 cells and ILC-2 in asthma’s pathogenesis, these two types of immune cells exhibit mechanistic similarities, with a prominent feature being the upregulation of the transcription factor GATA-3. This transcription factor stimulates the synthesis of Type 2 cytokines and chemokine receptors [31,32]. Among these cytokines, IL-5 regulates eosinophil development within the bone marrow [33]. To impact the underlying pathogenesis of airway eosinophilia, IL-5 signals eosinophil chemotaxis, ensuring their survival and subsequent cytokine release.

Additionally, IL-5 and IL-13 induce the production of other molecules, including eosinophil cationic protein, major basic protein, tumor necrosis factor, and eicosanoid pathway metabolites, all contributing to bronchial hyperreactivity. The elevated expression of IL-13 in the lungs, alongside IL-4, influences the signaling of ICAM-1 and VCAM-1 [33,34]. These molecules, released by Th-2 cells, prompt perivascular and peribronchial infiltrates to penetrate the lung interstitium, leading to bronchial smooth muscle hyperreactivity, chemokine induction, and epithelial injury.

Immune recognition of environmental allergens is instigated by antigen-presenting cells (APCs), including dendritic cells (DCs), B lymphocytes, and various other cell types. These cells trigger the maturation of naive T cells into Th-2 cells, capable of producing cytokines such as IL-4, IL-5, and IL-13 [6,27]. Among these, Th-2 cells generate IL-4 and IL-13, which stimulate the differentiation of B cells into plasma cells and shift antibody production to the IgE isotype [31]. The interplay between allergen-specific IgE molecules, the allergen itself, and effector cells such as mast cells and basophils governs the release of mediators such as histamine, tryptase, leukotrienes, and prostaglandins, all of which can trigger asthma symptoms [27]. Moreover, IL-4 contributes to the polarization of naive T cells toward Th2 cells, thus amplifying the overall Th-2-mediated inflammatory response [30,35].

Additional pertinent roles of IL-4 in asthma’s pathogenesis encompass the induction of VCAM-1 expression, which drives the migration of eosinophils, basophils, monocytes, and T cells to the site of allergic inflammation. Furthermore, IL-4 induces the expression of mucin genes, resulting in heightened production of airway mucus [36,37]. Intriguingly, even non-allergic individuals with eosinophilic airway inflammation exhibit elevated IL-5 levels (similar to allergic patients). This suggests that IL-5 is pivotal in determining eosinophilic airway inflammation, even without an allergic stimulus [38]. More recently, a second mechanism of MC upregulation has been elucidated, involving the synthesis of IL-9 by both Th-2 and ILC-2 cells [5,39]. In asthma, MCs migrate to a specific location in the airway epithelium and undergo significant changes in their protease profile, which may serve to regulate inflammation and can be therapeutically targeted [30,35]. Targeting these cells and their individual interactions with the airway epithelium, airway nerves, and other innate immune cells including eosinophils may be an effective treatment strategy for some patients [27]. Undoubtedly, ongoing research will reveal more about the origin of this mast cell population, the regulation of the trafficking of these cells to the airways, and the manner in which these cells interact with other cell populations in the airways [20,37].

IL-17 (comprising IL-17A and IL-17F) is a central cytokine in yet another adaptive immune system mechanism linked to asthma’s pathogenesis, particularly in neutrophilic phenotypes [40]. Th-17 cells, along with other T cells, NK cells, ILC-3, and mast cells, contribute to the production of this cytokine. The influx of neutrophils, driven by a surplus of IL-17A and IL-17F, is partially governed by the stimulation of airway epithelial cell and stromal cell cytokine production, thus influencing the chemotaxis of neutrophils to the site of inflammation. In the context of neutrophilic asthma, IL-17 is the principal signaling molecule that triggers the activation of its receptor. Moreover, this receptor is expressed in smooth muscle cells, leading to the hypertrophy of these cells [41]. Alongside its presence on epithelial fibroblasts, macrophages, and endothelial cells, the IL-17 receptor also finds expression on epithelial fibroblasts, providing insights into the potential role of IL-17 in airway remodeling [42]. Researchers posit that cytokines released by neutrophils—such as IL-6, TGF-beta, IL-1beta, TNF-alpha, IL-21, and IL-23—can activate crucial transcription factors such as STAT3 and RORC2. This activation promotes the differentiation of naive CD4+ T cells into Th-17 cells [43].

More than 60 genetic loci have been associated with asthma, with some of these loci also being linked to severe forms of the condition. Genome-wide association studies (GWAS) conducted on children and adults have pinpointed five loci connected to severe exacerbations and implicated multiple genes that participate in immune responses, such as IL33, IL1RL1, and CDHR3 [44]. Additionally, GWAS efforts have identified 24 loci linked to moderate-to-severe asthma [44,45]. The exploration of gene expression in persistent and severe asthma has been the focus of several studies. These investigations have encompassed a range of cell types, including airway epithelial cells, whole blood, sputum, and bronchoalveolar lavage. It is crucial to note that gene expression is inherently tissue-specific [46,47,48,49].

Furthermore, certain studies [50,51] have compared differences in gene expression responses to specific interventions. Predictably, various gene expression patterns have been uncovered, implying that diverse mechanisms contribute to the persistence and severity of asthma. Many of these mechanisms are intertwined with altered immune responses. Although sputum proteomics is a nascent field of study within asthma research, a handful of investigations have examined the intersection of proteomics and epigenetics in severe asthma [52,53,54,55].

## 3. Clinical and Molecular Phenotypes of Asthma

Numerous multicenter studies have been conducted to identify different asthma phenotypes [56,57,58,59]. The cohorts under investigation, the illness characteristics utilized in each clustering study, the computational methods employed, and the number of clusters identified during the research all exhibit considerable variation. Despite these distinctions, the findings of all studies indicate the presence of approximately four major phenotypes or endotypes of asthma in adults. These include early-onset mild allergic asthma, moderate-to-severe allergic remodeling asthma, late-onset allergic non-eosinophilic asthma, and late-onset allergic eosinophilic asthma [60].

In contrast, utilizing an unsupervised cluster analysis of the clinical and physiological characteristics of the condition [56], research has revealed five predominant phenotypes in adult patients: mild-early-onset allergic disease, moderate early-onset allergic disease, late-onset eosinophilic non-allergic disease, severe early-onset eosinophilic allergic disease, and late-onset non-allergic neutrophilic severe asthma with fixed airflow obstruction.

However, the heterogeneity of asthma is not confined to endotypes alone; clinical and inflammatory features (phenotypes) may also exhibit variability. Patients with similar clinical and inflammatory features can be categorized as belonging to a specific “phenotype” of asthma [4,7,61,62]. Comorbidities directly impacting asthma severity [63,64,65,66] and nutritional factors such as iron or vitamin D deficiency [67] further complicate and augment the heterogeneity of asthma’s clinical presentation.

**Figure 2:**
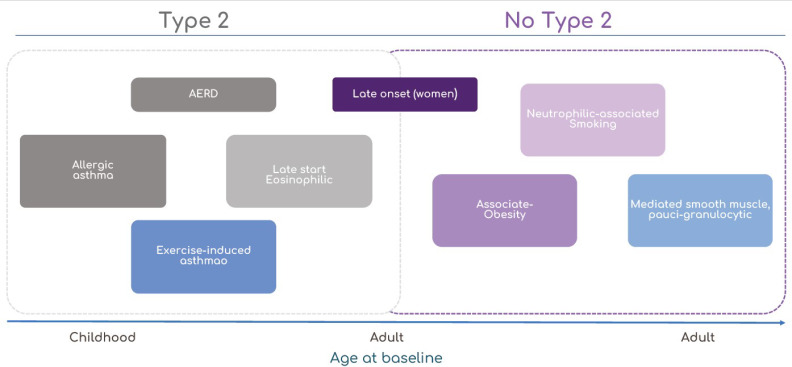
By applying statistical analyses to clinical, physiological, and laboratory characteristics, new subphenotypes and associated causal pathways, or endotypes, of asthma are being discovered. AERD = aspirin-exacerbated respiratory disease. Adapted from Wenzel, S. E. *Nat. Med.* [62].

Studies allude to an “endotypic range”, classified as complex Type 2 high or low, with one end of the spectrum being eosinophilic and the other being neutrophilic [68]. The existence of a “mixed” endotype as a potential presentation in inpatients has also been documented [69,70,71]. This “mixed” endotype may signify a convergence between these syndromes (Figure 2).

### 3.1. Mild-Early-Onset Allergic Asthma

First, allergic asthma is interconnected with other disorders, such as atopic dermatitis and allergic rhinitis, characterized by Th2 cell responses closely associated with allergic asthma and often initiated in childhood. This form of asthma stems from early-life exposure to environmental allergens such as house dust mites, pollen, cockroaches, or animal dander; however, it can also emerge later in life upon encountering a new allergen, such as one found in the workplace. Cytokines of Type 2, namely IL-4, IL-5, IL-9, and IL-13, are produced by allergen-specific Th2 cells upon allergen recognition [72]. These cytokines lead to the accumulation of numerous eosinophils in the airway wall, excessive mucus production, and the generation of IgE by allergen-specific B cells. These IgE antibodies can be detected in the serum or through a positive skin test.

Nonetheless, the early stages of infancy constitute a pivotal period for the immune system and lung structural development, and it is during this phase that the disease initially presents itself. Neonatal development shapes both lifelong homeostasis and susceptibility to immune-mediated conditions such as asthma. Consequently, alterations in the pulmonary environment during this “window of opportunity” can bring about changes in immune cell and organ behavior that persist long after the initial trigger has subsided [73,74].

Early-onset eosinophilic asthma is often linked with a family history of atopy and carries a clearly defined prognosis responsive to steroids [72]. Nevertheless, an allergic severe asthma phenotype that shows poor responsiveness to corticosteroids has been delineated and appears to be one of the most-prevalent severe asthma manifestations [75,76].

### 3.2. Late-Onset Eosinophilic Asthma

In contrast to allergic asthma, nonallergic asthma typically manifests later in life, is more prevalent among obese and female patients, and poses considerable challenges in terms of treatment [77]. The late-onset asthma phenotypes are divided into two categories: T2 and non-T2. The non-T2 variant is associated with smoking, advancing age, and obesity. On the other hand, the T2-associated type may present with elevated airway eosinophil counts, recurrent and chronic rhinosinusitis with nasal polyps (CRSwNP), aspirin sensitivity, and other symptoms [78].

Asthma endotypes, categorized as T2 (mostly eosinophilic) and non-T2 (non-eosinophilic, occasionally neutrophilic, and metabolic), have emerged from recent efforts to classify asthma phenotypes [79].

Patients with late-onset asthma are often marked by persistent airway eosinophilia and a limited response to corticosteroid treatment [72,80]. While the precise mechanism of disease progression in adult-onset eosinophilic asthma remains incompletely elucidated, this phenotype typically features ILC-2-driven inflammation orchestrated by cytokines such as IL-5 and IL-13 [81]. Consequently, this pattern culminates in profound eosinophilic airway and systemic inflammation.

#### 3.2.1. Non-Steroidal Anti-Inflammatory-Drug-Exacerbated Respiratory Disease 

Recognized as also as aspirin-exacerbated respiratory disease (AERD), this is a prominent severe asthma phenotype emerging in adulthood [82]. Non-steroidal anti-inflammatory-drug-exacerbated respiratory disease (N-ERD) is often linked with polymorphisms in the genes encoding prostaglandin E2 receptor 2 and cysteinyl leukotriene receptor 1 [83]. Pathological pathways indicate an escalation in Cys-LTs’ production, underscoring a disrupted arachidonic acid metabolism that likely contributes to disease progression [82,83]. Functioning as a T2 airway inflammatory disorder, it is characterized by heightened peripheral and sputum eosinophilia, a high prevalence of coexisting CRSwNP, and hypersensitivity to non-steroidal anti-inflammatory drugs [82]. Indicative biomarkers for N-ERD encompass elevated blood and sputum eosinophil counts, potentially linked to heightened serum IgE levels, and, most notably, an increased presence of Leukotriene E4 (LTE4) [72].

#### 3.2.2. Exercise-Induced Bronchoconstriction 

An inherent trait of the traditional understanding of asthma, excessive exercise triggers an evaporative force on epithelial cells, leading to water loss and the release of pro-inflammatory mediators into the interstitium. This, in turn, prompts mast cell degranulation [84]. The secretion of cytokines and signaling molecules by mast cells leads to hyperreactivity and structural changes within the airways. Moreover, pro-inflammatory mediators discharged by epithelial cells prompt bronchoconstriction [85].

Symptoms of exercise-induced bronchoconstriction (EIB) intermittently intensify during physical activity, a phenomenon that has been scrutinized with particular attention in preschool- and school-age populations. This phenotype in the pediatric demographic has been demonstrated to have a genetic predisposition attributable to a polymorphic impairment of aquaporin channel function, which results in tissue dehydration. EIB forms part of the Th-2-cytokine-induced pathway, coupled with the dysregulation of the arachidonic acid metabolism pathway [86,87,88].

### 3.3. Non-Eosinophilic Neutrophilic Asthma Phenotypes

Clinicians have identified two phenotypic forms that share a common genetic and molecular mechanism of pathogenesis. The initial form of non-atopic neutrophilic asthma is known as paucigranulocytic asthma [89], characterized as a typically benign asthma variant with no detectable airway inflammation. The second type involves patients with an elevated neutrophil count and is termed the “non-atopic neutrophilic asthma” phenotype. This presentation predominantly occurs in adults and exhibits varying degrees of disease severity [90,91].

This phenotype is thought to be orchestrated by the immune cell Th-17, polymorphic alterations influencing the aberrant mRNA expression of the *NLRP3* gene—the producer of cryopyrin, an inflammasome—and an upsurge in the expression of IL-1 beta [91]. Additionally, within bronchoalveolar lavage, an elevated concentration of matrix metalloproteinase-9 (MMP-9) is observed. MMP-9 functions as a regulatory protein for neutrophil transendothelial migration, thereby indicating non-atopic neutrophilic asthma.

Smoking, obesity, and age are customary clinical conditions linked to neutrophilic inflammation in asthma [72]. Among smokers, asthma takes on a mixed inflammatory pattern, displaying a stronger propensity for neutrophilia over eosinophilia. In this scenario, Th-17-driven neutrophilic inflammation is marked by oxidative stress, prompting substantial airway remodeling [92]. This phenotype involves a series of oxidative-stress-induced pathways accompanied by elevated leptin levels [93].

Neutrophilic asthma, prevalent among the elderly, represents a steroid-resistant variant of the condition, classified as an immunosenescent inflammatory disorder driven by Th-17 cells [94,95]. While the exact pathological mechanisms remain unclear, investigations propose that the respiratory system experiences reduced functionality due to inflammation-triggered airway remodeling [72,94,96].

#### 3.3.1. Obesity-Related Asthma

Numerous genetic variations linked to obesity-related asthma have been pinpointed in research studies, encompassing ADIPOQ [97,98], retinoid-related orphan receptor C (RORC), IL17A [99], TNF-α [100,101,102], beta-2 adrenergic receptor (ADRB2) [103], and IL-6 [104]. This form of asthma, associated with obesity, is influenced by gender and is more prevalent among females. Compared to other phenotypes, this manifestation is characterized by heightened activation of the innate immune system, particularly involving the immune pathways governed by ILC-2 cells [105].

Elevated levels of IL-6, an endogenous pro-inflammatory cytokine for neutrophils, correlate with increased neutrophil counts in cases of obesity-related asthma [104,106]. Similarly, observations have revealed reduced levels of adiponectin [93] in obese asthma patients, aligning with poorer lung function outcomes [107,108]. Consequently, elevated serum IL-6 levels are potential biomarkers for obesity-related asthma [72].

Castro-Rodriguez et al. [109] reported, for the first time, that obesity was related to the incidence of wheezing in girls, but not in boys. However, it was true only among girls with early menarche (<11 years). After adjusting for confounders, e.g., skin test and parental BMI, girls who became overweight (BMI ≥ 85 to <95 percentile) or obese (≥95 percentile) between 6 and 11 years of age were more likely to develop new infrequent (aOR: 6.8; 95% CI [2.4–19.4], *p* = 0.0001) and frequent (aOR: 5.5; [1.3–23.3], *p* = 0.015) wheezing episodes than those who did not become overweight or obese. Later, Guerra et al. [110] in the same cohort found that obesity and early onset of puberty were independent risk factors (aOR: 8.9; [1.7–46.8] and aOR: 0.64; [0.44–0.93], respectively) for the persistence of asthma during adolescence (up to 16 years of age), with a trend for girls. A review [111], including eleven epidemiologic worldwide studies, supports the thesis that obese girls who have an early menarche (<11 years of age) constitute a new asthma phenotype in childhood.

Managing obesity-related asthma can pose greater challenges than in non-obese patients, including children [112]. The surplus weight can impede the effective delivery of medications to the airways [113], potentially diminishing the efficacy of inhaled corticoid treatments [114]. Furthermore, individuals with obesity might necessitate higher medication doses to attain sufficient control over asthma symptoms. Effectively addressing obesity-related asthma entails a comprehensive approach that targets both the obesity and asthma components [115].

Typically, treatment involves a blend of lifestyle adjustments, encompassing weight management, regular physical activity, and a balanced diet, in conjunction with appropriate asthma medications [114]. For children grappling with obesity-related asthma, early intervention, education, and support play pivotal roles in successful management [114]. By concurrently addressing both obesity and asthma, the potential exists to enhance asthma control, alleviate symptoms, and improve the overall well-being of afflicted patients [116,117].

#### 3.3.2. Perimenstrual Asthma

There is no clear definition of perimenstrual asthma (PMA) in the literature. In general, worsening asthma during the luteal phase and/or the first days of menstruation is characterized by worsening lung function tests. PMA is a distinct, highly symptomatic, and exacerbation-prone asthma phenotype distinguished by aspirin sensitivity, less atopy (lower IgE level), and lower forced vital capacity compared to traditional allergic asthma. In population-based studies, rates of hospitalization for asthma are similar by sex in the early adolescent years [118], but three-times higher in women aged 20–50 years. After menopause, the prevalence of asthma decreases and returns to male levels [119,120]. Sex hormones are powerful immune and inflammatory modulators [121]. Estrogens have a significant impact on the onset and/or progression of several autoimmune diseases, as well as bacterial and parasitic infectious processes [121,122,123,124]. They act via the estrogen receptors (ER) alpha and beta [120,121,125], which are expressed by a variety of immune cells [126]. MCs are thought to be the primary players in the clinical scenario of inflammation and pain [120,127,128]. MCs are found in the endometrium and myometrium and are mostly found in the basal layer [129]. MCs are upregulated in the tissue in response to a variety of stimuli, including neurogenic factors, fluctuating estrogen levels, and menstrual blood [130]. When activated, MCs degranulate and release a variety of inflammatory mediators that aid in the maintenance of the immune response [129]. Sex hormones control MCs’ functionality and distribution in a variety of tissues [130,131,132,133], both physiologically and pathologically. In this regard, a link has been proposed between female sex hormones, MCs, and the development of asthma and allergies [120,134]. Furthermore, the presence of sex steroid receptors on MCs suggests that sex hormones may exert biological effects through binding to these receptors [135]. Exogenous estrogen and progesterone have both been shown in studies to play a role in PMA management [120,136]. In a prospective study of 106 postpubertal women with asthma, those who used oral contraception (OC) had lower asthma symptoms, better pulmonary function, and better asthma control than those who did not [137]. Tan et al. contrasted the airway reactivity to adenosine monophosphate (AMP) in female asthmatics with regular menstrual cycles and those taking an OC (21 days of active treatment and a 7-day break). The group with natural menstrual cycles had a significant increase in airway reactivity during the luteal phase, which coincided with an increase in progesterone and estradiol [138].

## 4. Therapeutic Targets in the Era of Personalized Medicine

Standard anti-inflammatory drugs, primarily inhaled corticosteroids (ICSs) and bronchodilators, remain the primary methods for treating asthma. However, these approaches do not consider the diverse range of asthma traits and endotypes [11,116], as recommended by multiple guidelines [76,139,140,141,142,143,144]. While this “one size fits all” strategy may be effective for the majority of patients, approximately 5–10% of individuals with asthma do not achieve clinical or functional control even with high doses of ICSs, along with other controllers and/or prolonged use of oral corticosteroids (OCSs). These individuals are categorized as severe asthmatics [144]. For them, a more-personalized and -precise therapeutic strategy is essential, involving the identification of distinct phenotypes and endotypes to target specific underlying factors. In this regard, we outline the key methods for treating asthma based on its various endotypes [61,145].

### 4.1. Therapeutic Strategies for Anti-IgE

Omalizumab, an anti-IgE monoclonal antibody utilized to manage severe allergic asthma, marked the advent of biologically targeted asthma treatments [146]. Mechanistically, this drug binds to IgE and hinders its interaction with downstream receptors, curtailing its activity. Administered subcutaneously, Omalizumab is delivered in varying doses (ranging from 75 to 375 mg) contingent upon the patient’s pretreatment serum IgE level [147]. Principally, the inhibition of IgE leads to a reduction in airway inflammation, an escalation in eosinophil apoptosis, and a decrease in IgE receptors on basophils and mast cells, ultimately mitigating the release of mediators. The impediment of IgE also thwarts airway remodeling by curtailing the secretion of growth factors from epithelial and smooth muscle cells within the airway [148]. Lastly, the anti-IgE property retards T cell maturation, resulting in diminished IgE production by plasma cells [149]. For over a decade, Omalizumab has remained the sole biologic drug on the market for asthma treatment, primarily showcasing its efficacy in managing the severe allergic phenotype of asthma. It achieves this by reducing exacerbation rates and improving patients’ quality of life [146].

Numerous real-world studies have emerged over eighteen years of utilizing Omalizumab, shedding light on its effectiveness [150]. These investigations have underscored that omalizumab notably diminishes the frequency of exacerbations and is a preventive measure against their occurrence [151,152,153,154,155,156]. Furthermore, it enhances asthma control by mitigating daily symptoms, reducing activity constraints [153], and lessening rescue medication requirements [154,156]. Remarkably, healthcare resources, encompassing emergency room visits and hospitalizations for lung function concerns, have also demonstrated improvement [150,157,158,159].

### 4.2. Therapeutic Strategies Involving Anti-IL5

Interleukin-5 (IL-5) is pivotal in sustaining eosinophilic inflammation across all Type 2 asthma phenotypes. Recently, novel biologic medications that target this cytokine have emerged as therapeutic options for individuals with severe eosinophilic asthma [33]. Mepolizumab, a monoclonal antibody, obstructs circulating eosinophils’ proliferation, maturation, and survival [160]. This intervention has proven effective in averting asthma exacerbations [10], elevating asthma-related quality of life [161] and reducing the necessity for OCS treatment [162], potentially mitigating the risk of OCS-related adverse events [163,164]. Administered subcutaneously every 4 weeks at a fixed dose of 300 mg, Mepolizumab’s use is currently indicated for patients with severe eosinophilic asthma and blood eosinophil counts surpassing 300/mcc [160,165].

Reslizumab, another monoclonal antibody targeting IL-5, contrasts with Mepolizumab in terms of administration. Reslizumab is given intravenously, and its dose varies (ranging from 100 to 575 mg) based on the patient’s weight [166]. Reducing the risk of asthma exacerbations among treated patients positively impacts their overall quality of life [167].

The third target within the IL-5 cascade operates through a distinct mechanism: Benralizumab, an IL-5-receptor-alpha-targeting monoclonal antibody [168]. The inhibition of IL-5 receptor alpha triggers an antibody-dependent cellular cytotoxicity mechanism mediated by NK cells against eosinophils and basophils [168,169]. Similarly, Benralizumab yields favorable outcomes in treated patients, including diminished asthma exacerbations [12], decreased OCS utilization, and improved quality of life [170,171].

### 4.3. Therapeutic Strategies for Anti-IL4-Receptor Alpha

Dupilumab, an IgG4 monoclonal antibody, specifically targets the IL-4R alpha chain, a component of the shared receptor for the pro-inflammatory cytokines IL-4 and IL-13. This dual functionality effectively hinders Type-2-cytokine-driven asthmatic inflammation [172]. Presently, the drug is authorized for managing atopic dermatitis [173,174,175], serving as an add-on maintenance therapy for individuals aged 6 years and older with moderate-to-severe asthma, exhibiting either an eosinophilic phenotype or dependency on OCSs [176,177]. Dupilumab also finds approval for CRSwNP [178,179] and eosinophilic esophagitis (EoE) [180,181]. Clinical trials evaluating dupilumab’s efficacy in asthma underscore a reduction in asthma exacerbation rates [14], attributed to substantial lung function enhancement and favorable tolerability, including glucocorticoid withdrawal [177,182,183]. Recent data showcasing dupilumab’s positive impact on CRSwNP outcomes render it an effective choice for the concurrent treatment of Type 2 diseases [184]. Dupilumab’s consistent effectiveness and safety span all age groups, extending even to pediatric patients [185,186,187,188]. This comprehensive profile has earned the biologic the most approvals from regulatory agencies for pediatric use.

Trials assessing the efficacy of dupilumab in asthma encompass placebo-controlled, phase 3 or 2b trials spanning 24 to 52 weeks of treatment in patients aged 12 years with moderate-to-severe asthma [13,182]. Adding subcutaneous dupilumab (200 or 300 mg every 2 weeks) to background therapy was generally well-tolerated in these investigations. This incorporation reduced the frequency of severe asthma exacerbations, improved lung function, enhanced asthma control, and, where specified, resulted in a better health-related quality of life (HR-QOL). Moreover, it enabled the reduction of OCS maintenance doses without compromising asthma control. Dupilumab consistently demonstrated effectiveness across various patient subgroups; however, individuals with heightened Type 2 immune activity, such as elevated eosinophil counts and fractional exhaled nitric oxide levels, typically observed a more-pronounced therapeutic advantage [172].

### 4.4. Therapeutic Strategies against TSLP

Tezepelumab is an IgG monoclonal antibody specifically designed to inhibit TSLP. This inhibition targets the interaction between the TSLP protein and its receptor complex [25], reducing the recruitment of APCs for the maturation of adaptive immune cells. Consequently, there is an overall suppression of Type 2 inflammation [25,105,189,190].

In recent clinical trials, the administration of Tezepelumab demonstrated notable effects. Notably, there was a decrease in blood eosinophil count, IgE levels, and fractional exhaled nitric oxide (FeNO) levels compared to baseline measurements [191,192]. Tezepelumab-treated individuals with asthma exhibited a significant clinical reduction in exacerbation episodes and decreased the need for OCSs [193]. Moreover, the treatment resulted in fewer hospitalizations and emergency department visits [169], along with improved lung function and quality of life [189,194].

Combining two biologics for severe asthma, also known as biologic combination therapy, holds the potential to address different aspects of the disease’s complex immunopathology and provide enhanced therapeutic benefits. Severe asthma often involves various inflammatory pathways, and targeting multiple pathways simultaneously with biologic medications could lead to improved symptom control and quality of life for patients [195]. The idea of biologic combination therapy has gained attention, and some clinical trials are exploring this approach for severe asthma. However, more research is needed to establish the safety, efficacy, and long-term outcomes of combining different biologics in this context [195]. Ultimately, personalized treatment plans based on a patient’s unique immunopathology will guide decisions regarding biologic monotherapy or combination therapy for severe asthma.

The failure of treatment in asthma, particularly with biologic medications, can result from a variety of factors, including the choice of treatment mechanism and the development of autoantibodies against the biological treatment [141]. To address these challenges, ongoing research is focused on refining patient selection criteria, identifying reliable biomarkers, developing combination therapies, and improving our understanding of asthma’s molecular mechanisms. It is important for healthcare providers to closely monitor treatment response, assess any changes in disease phenotype, and consider adjustments to treatment strategies as needed to achieve optimal outcomes for patients with severe asthma [76,139,142].

## 5. Tools That Use Machine Learning to Improve Asthma Care in the Clinic

Comprehensive multi-omics datasets encompassing genomic/epigenomic, transcriptomic, proteomic, metabolomic, and lipidomic profiles have become publicly available, coupled with clinical information. This wealth of data allows one to delve deeper into molecular phenotypes and their correlations with asthma traits [196]. In certain instances, these genetic traits can evolve into endotypes when their association with distinct disease outcomes is established through treatment strategies targeting specific pathways. However, the transition from genetic traits to endotypes necessitates rigorous testing, a step that remains pending.

As demonstrated, amalgamating the multi-omics characteristics within a single individual can unveil crucial insights that would remain obscured when examining each data type in isolation. Moreover, it is imperative to contemplate incorporating and integrating clinical data, enabling a comprehensive examination while considering pivotal factors. The convergence of multi-omics and machine learning methodologies offers diverse avenues for exploration [196,197].

Omics technologies, encompassing genomics, transcriptomics, proteomics, metabolomics, and other innovative methodologies, have witnessed a growing application within asthma research. Their utilization extends to the unbiased identification of potential biomarkers [197]. By leveraging these technologies, asthma can be scrutinized from many perspectives, facilitating a more-holistic grasp of the intricate and multifaceted nature of the disease [197,198].

To illustrate, genomics plays a pivotal role in the identification of genetic risk factors, as well as potential therapeutic targets for asthma [198]. In contrast, transcriptomics offers valuable insights into gene expression patterns linked to asthma, thereby enhancing our comprehension of the disease’s underlying pathophysiology [199]. Furthermore, proteomics and metabolomics unveil alterations in protein and metabolite levels, respectively, which hold promise as potential asthma biomarkers [198]. Beyond this, the amalgamation of multi-omics data with comprehensive phenotyping and clinical outcomes presents a route to attaining profound functional insights into intricate conditions such as asthma [200]. This methodical integration of omics information, leveraging data from diverse sources and patient populations, is critical in untangling the clinical intricacies and the origins of asthma [50,200].

Nonetheless, it is crucial to acknowledge that, while omics technologies have narrowed the gap between laboratory research and patient care, several design and methodological hurdles remain to be surmounted before the full integration of omics into asthma patient management [51,199]. Despite these challenges, the potential of omics in shaping the future of asthma care is promising, with the capacity to elevate precision treatment for individuals with asthma [51,200]. Furthermore, recent advancements have made deep learning methods, once intricate and opaque, more comprehensible. Techniques such as backpropagation have rendered them more accessible, enabling inferences beyond mere prediction. These approaches can amalgamate diverse multi-omics datasets, unearthing patterns within asthma’s molecular traits. Such patterns can subsequently be subjected to hypothesis-driven studies and related perturbation systems to delineate distinct endotypes [197]. The careful selection of an appropriate methodology that considers these aspects paves the way for robust scientific findings that can be replicated and effectively utilized. Additionally, transcriptomics offers insights into the gene expression patterns underpinning asthma, enriching our grasp of the disease’s pathophysiology [199].

Epigenomics, for its part, can unveil alterations in DNA methylation, histone modification, and other epigenetic markers that could influence gene expression, thereby contributing to the emergence and progression of asthma [197,199,200]. Correspondingly, metabolomics can spotlight shifts in metabolite levels, holding the potential to function as noteworthy asthma biomarkers [50]. Through the harmonious amalgamation of data emanating from these diverse omics strategies, investigators can foster a more-holistic comprehension of the intricate biological processes intrinsic to asthma [199]. This holistic insight, in turn, facilitates the identification of distinctive asthma endotypes—subcategories of asthma characterized by unique pathophysiological mechanisms [50]. The comprehension of these endotypes plays a pivotal role in charting the course for personalized treatment strategies, custom-tailored to the precise requirements of individual patients [50,51,200].

Furthermore, harmonizing multi-omics data with thorough phenotyping and clinical outcomes presents an avenue to access more-profound functional insights into intricate maladies such as asthma. This methodical fusion of omics data, harnessing information from diverse ethnic backgrounds gleaned from various sources, can furnish invaluable revelations to untangle the intricate clinical aspects and origins of asthma [50,51,198,199,200]. Omics data can be vast and complex, making their interpretation challenging. Integrating different omics datasets and identifying meaningful patterns requires advanced computational methods, and the results may not always be straight-forward. While omics studies generate valuable insights, translating these findings into effective therapies can be challenging. Developing drugs targeting specific molecular pathways identified by omics approaches requires extensive validation and clinical trials.

## 6. Gene and Cell Therapy

Mesenchymal stromal cells (MSCs) have garnered attention as a promising alternative for treating respiratory diseases due to their beneficial properties, including anti-inflammatory, antiapoptotic, antimicrobial, and antifibrotic attributes [201]. Nonetheless, despite the theoretical potential of these cells, the primary results from clinical trials investigating MSCs for respiratory disorders have fallen short of anticipated expectations [202,203].

An avenue for enhancing the favorable impacts of MSCs involves genetic manipulation. This strategy encompasses the modulation of genes engaged in cell survival pathways and immunomodulation, achieved through plasmid transfection, transduction via viral vectors, or employment of miRNA and small interfering RNA [204]. For instance, in a model of lung disease, the elevation of the Developmental Endothelial Locus-1 gene in MSCs obtained from murine bone marrow resulted in diminished lung injury histopathological indices, reduced pulmonary edema, fewer neutrophil counts, lowered TNF-α levels, decreased protein concentration in bronchoalveolar lavage fluid (BALF), and reduced myeloperoxidase activity in lung homogenates [204,205].

Nonetheless, converting MSC transplantation into an effective procedure poses a considerable challenge. Despite the substantial volume of experimental evidence endorsing diverse strategies for enhancing MSC functionality, including genetic manipulation, a substantial journey remains before these methodologies transition from the laboratory setting to practical clinical application [205].

Genetically modified cells have secured approval and are presently undergoing utilization in early-phase clinical investigations targeting patients with pulmonary hypertension, exemplified by the Pulmonary Hypertension and Angiogenic Cell Therapy (PHACeT) trial [206]. Nevertheless, further research is imperative to comprehensively grasp the potential and constraints of employing genetically modified MSCs for addressing respiratory diseases.

## 7. Conclusions

Asthma is a complex, multifactorial ailment that presents divergently across distinct patient subsets, resulting from the diverse expression of inflammatory pathways encompassing innate and adaptive immune systems. The simplistic notion of asthma as a unifying disorder characterized by chronic airway inflammation, bronchial hyperreactivity, airway obstruction, and structural airway modifications is no longer tenable. In light of this understanding, a more-individualized strategy toward asthmatic patients is imperative. This entails the integration of precision medicine, facilitating the finer delineation of patients into phenotypes and endotypes, and the tailored selection of optimal medications for each individual, a “personalized treatment” paradigm that is particularly crucial for individuals afflicted by severe asthma.

Identifying phenotypes necessitates meticulous evaluation across various clinical facets (e.g., atopy presence, comorbidities, clinical presentations), lung function patterns (e.g., bronchial reversibility extent, fixed airway obstruction presence, airway hyperreactivity degree), and engagement of sputum and systemic inflammatory factors (e.g., eosinophilic, neutrophilic, mixed). The precision medicine framework for asthma marks a wholly innovative paradigm, offering enhanced prospects for more-efficacious and -fitting patient interventions and novel revelations concerning the immunological dimensions of asthma warranting deeper investigation.

## Data Availability

Not applicable.
